# Dynamic cerebral autoregulation across the cardiac cycle during 8 hr of recovery from acute exercise

**DOI:** 10.14814/phy2.14367

**Published:** 2020-03-12

**Authors:** Joel S. Burma, Paige Copeland, Alannah Macaulay, Omeet Khatra, Alexander D. Wright, Jonathan D. Smirl

**Affiliations:** ^1^ Concussion Research Laboratory Faculty of Health and Exercise Science University of British Columbia Kelowna BC Canada; ^2^ Sport Injury Prevention Research Center Faculty of Kinesiology University of Calgary Calgary AB Canada; ^3^ Human Performance Laboratory Faculty of Kinesiology University of Calgary Calgary AB Canada; ^4^ Hotchkiss Brain Institute University of Calgary Calgary AB Canada; ^5^ Faculty of Medicine University of British Columbia Vancouver BC Canada; ^6^ MD/PhD Program Faculty of Medicine University of British Columbia Vancouver BC Canada; ^7^ Experimental Medicine Program Faculty of Medicine University of British Columbia Vancouver BC Canada; ^8^ Southern Medical Program University of British Columbia Kelowna BC Canada; ^9^ Alberta Children’s Hospital Research Institute University of Calgary Calgary AB Canada; ^10^ Libin Cardiovascular Institute of Alberta University of Calgary Calgary AB Canada

**Keywords:** acute recovery, blood pressure, cerebral autoregulation, cerebral blood flow, exercise

## Abstract

Current protocols examining cerebral autoregulation (CA) parameters require participants to refrain from exercise for 12–24 hr, however there is sparse objective evidence examining the recovery trajectory of these measures following exercise across the cardiac cycle (diastole, mean, and systole). Therefore, this study sought to determine the duration acute exercise impacts CA and the within‐day reproducibility of these measures. Nine participants performed squat–stand maneuvers at 0.05 and 0.10 Hz at baseline before three interventions: 45‐min moderate‐continuous exercise (at 50% heart‐rate reserve), 30‐min high‐intensity intervals (ten, 1‐min at 85% heart‐rate reserve), and a control day (30‐min quiet rest). Squat–stands were repeated at hours zero, one, two, four, six, and eight after each condition. Transcranial doppler ultrasound of the middle cerebral artery (MCA) and the posterior cerebral artery (PCA) was used to characterize CA parameters across the cardiac cycle. At baseline, the systolic CA parameters were different than mean and diastolic components (ps < 0.015), however following both exercise protocols in both frequencies this disappeared until hour four within the MCA (ps > 0.079). In the PCA, phase values were affected only following high‐intensity intervals until hour four (ps > 0.055). Normalized gain in all cardiac cycle domains remained different following both exercise protocols (ps < 0.005) and across the control day (*p* < .050). All systolic differences returned by hour six across all measures (ps < 0.034). Future CA studies may use squat–stand maneuvers to assess the cerebral pressure–flow relationship 6 hr after exercise. Finally, CA measures under this paradigm appear to have negligible within‐day variation, allowing for reproducible interpretations to be drawn.

## Graphical abstract

Nine participants performed squat‐stand maneuvers at 0.05 and 0.10 Hz at baseline before three interventions: 45‐min moderate‐continuous exercise (at 50% heart‐rate reserve), 30‐min high‐intensity intervals (ten, one minute at 85% heart‐rate reserve), and a control day (30‐min quiet rest). Squat‐stands were repeated at hours zero, one, two, four, six, and eight after each condition. exercise was shown to affect dynamic CA into recovery up to 4 hr following exercise, with HIIT demonstrating greater affects than MICT (each phase of the cardiac cycle responded differently to exercise, with the systolic component showing impairments for up to 4 hr, compared to diastolic and mean which recovered after 2 hr in all measures).

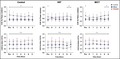

## 1 INTRODUCTION

The brain is the most highly perfused organ in the body. Although it comprises only 1.5%–2% of total body mass, it consumes roughly 10%–15% of resting cardiac output and 15%–20% of total oxygen consumption at rest (Williams & Leggett, 1989). Maintaining oxygen supply and nutrient delivery to cerebral tissue is therefore of critical importance, both at rest and during exercise, as the brain has limited substrate storage (Willie, Tzeng, Fisher, & Ainslie, 2014). Cerebral blood flow velocity (CBV) has been shown to increase 10%–20% during moderate intensity exercise (~60%–70% of an individual's maximal oxygen uptake; VO_2max_), as a result of parallel increases of neuronal activity, metabolism, and regional CBV (Marsden et al., 2012; Ogoh & Ainslie, 2009, 2009; Smith & Ainslie, 2017). During more intense exercise, CBV has been shown to either plateau or decrease, with the extent of reduction associated with the degree of hyperventilation‐induced cerebral vasoconstriction (Larsen, Rasmussen, Overgaard, Secher, & Nielsen, 2008; Ogoh & Ainslie, 2009; Smith & Ainslie, 2017; Subudhi et al., 2011).

The brain is able to regulate its blood supply somewhat independently of the rest of the body through a process known as cerebral autoregulation (CA) (Lassen, 1959; Paulson, Strandgaard, & Edvinsson, 1990; Willie et al., 2014). This has been previously quantified in two domains, which are thought to be on a spectrum: static (Lassen, 1959) and dynamic (Aaslid, Lindegaard, Sorteberg, & Nornes, 1989). The former refers to a technique where two steady‐state measurements are taken of CBV prior to and following blood pressure manipulation; whereas, the latter is in reference to the dynamic frequency range where CA is operant (<0.20 Hz) (Claassen, Levine, & Zhang, 2009; Zhang, Zuckerman, Giller, & Levine, 1998). Furthermore, dynamic CA has been examined using various analytic techniques during exercise (Bailey et al., 2011; Brys, Brown, Marthol, Franta, & Hilz, 2003; Ogoh, Dalsgaard, et al., 2005; Ogoh, Fadel, et al., 2005; Smirl, Hoffman, Tzeng, Hansen, & Ainslie, 2016; Tsukamoto et al., 2019) as well as during *immediate recovery (*<60 min*)* following exercise at mild (Ogoh, Fisher, et al., 2007), moderate (Ogoh, Fisher, et al., 2007; Willie, Ainslie, Taylor, Eves, & Tzeng, 2013), and heavy aerobic exercise levels (Ogoh, Fisher, et al., 2007; Tsukamoto et al., 2019). Conflicting findings have emerged from these aforementioned studies as several found CA remained intact following exercise when examining the relationship between arterial pressure and middle cerebral artery blood velocity (MCA) (Brys et al., 2003; Ogoh, Fisher, et al., 2007; Smirl et al., 2016; Willie et al., 2013); whereas, other studies found impairments in CA (Ogoh, Fisher, et al., 2007). For example, Ogoh and colleagues (2007) additionally examined the diastolic and systolic relationship between these two variables, finding alterations in the diastolic component following exercise (<10 min). There are three major limitations to these previous studies: (a) only the short‐term effects of CA during exercise or within 60 min of exercise cessation were examined; the duration of dynamic CA alterations postexercise remain unknown; (b) only two studies have examined the CA relationship across the cardiac cycle (Ogoh, Fadel, et al., 2005; Ogoh, Fisher, et al., 2007); as recent research has shown each phase of the cardiac cycle responds differently to a given stimulus, examining the mean may not accurately portray important subtleties between diastolic and systolic components (Smirl, Wright, Ainslie, Tzeng, & Donkelaar, 2018; Wright, Smirl, Bryk, & Donkelaar, 2018); and (c) only one of these prior studies employed a research method (oscillatory lower body negative pressure *during* the exercise intervention) which evoked sufficient coherence to provide reproducible interpretations of the associated phase and gain metrics (Smirl, Hoffman, Tzeng, Hansen, & Ainslie, 2015). Moreover, recent research has demonstrated when squat–stand maneuvers are employed to quantify CA parameters, coherence values are enhanced to near linear levels (~0.99) (Kostoglou et al., 2016; Smirl, Haykowsky, et al., 2014; Smirl et al., 2015, 2016, 2018; Smirl, Lucas, et al., 2014; Smirl, Tzeng, Monteleone, & Ainslie, 2014; Wright, Smirl, Bryk, & Donkelaar, 2018; Wright et al., 2018, 2018). This methodological approach leads to greater reproducibility within the CA outcome measures (phase, absolute gain, and normalized gain) (Claassen et al., 2009; Smirl et al., 2015), which will enhance the interpretability and application of the findings related to the current investigation.

Furthermore, the current dogmatic testing paradigm when examining cerebrovascular function is to require individuals in scientific investigations to refrain from exercise for at least 12 hr (Ainslie, Barach, et al., 2007; Ainslie, Hamlin, Hellemans, Rasmussen, & Ogoh, 2008; Smirl et al., 2016), 24 hr (Fisher, Ogoh, Young, Raven, & Fadel, 2008; Gelinas et al., 2012; Ogoh, Brothers, et al., 2005; Ogoh, Dalsgaard, Secher, & Raven, 2007; Ogoh, Dalsgaard, et al., 2005; Ogoh, Fadel, et al., 2005; Ogoh, Sato, et al., 2010), or 48 hr (Bailey et al., 2011) before the assessment period to limit potential effects of exercise on dynamic CA outcome metrics. To the best of the authors’ knowledge, these time constraints were not determined by empirical observation. There is a paucity of data in the broader literature to provide a sufficient objective window indicating when CA returns to typical resting levels following aerobic exertions. Therefore, the aim of this study, was to assess the dynamic CA response with augmented coherence via squat–stand maneuvers, before and for an 8‐hr‐period immediately after acute bouts of exercise. This study assessed two types of exercise, namely moderate intensity continuous exercise (MICT) and high intensity interval training (HIIT). Two different modalities of exercise were performed as the former is known to cause cerebral vasodilation, whereas the latter results in cerebral vasoconstriction (Smith & Ainslie, 2017). Therefore, as these protocols result in different hemodynamic responses, both were used to examine the pressure‐flow recovery time course following exercise, as they elicit divergent physiological modulations. A control rest day was also used to determine the variability of the dynamic CA response across a typical work day (Ainslie, Murrell, et al., 2007). Furthermore, the relationship between blood pressure (BP) and CBV was assessed separately for diastolic, mean, and systolic components of the cardiac cycle. This was done to examine which aspect of the cardiac cycle dynamic CA is most susceptible to effects of exercise, as previous studies have demonstrated exercise dissimilarly affects each cardiac cycle phase (Ogoh, Dalsgaard, et al., 2005; Ogoh, Fisher, et al., 2007). It was hypothesized exercise would reduce the effectiveness of CA (i.e., a decreased phase and an increased gain) for at least 1 hr after exercise with the greatest effects occurring in the diastolic phase of the cardiac cycle based on prior observations indicating diastolic pressure waves pass through the cerebrovasculature relatively unimpeded, while systolic oscillations are more effectively buffered and less likely to be influenced by the exercise modality (Smirl et al., 2018). Moreover, these alterations were anticipated to occur irrespective of the exercise intensity, with phase and gain values returning back to baseline at hour one following exercise.

## 2 METHODS

### 2.1 Study design

**Figure 1:**
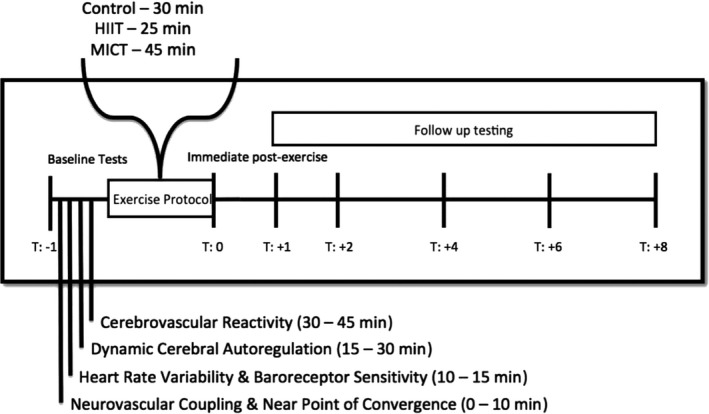
Representative trace of the testing order of the cardiovascular and cerebrovascular measures day before engaging in a randomly selected exercise condition. Each serial follow‐up measure post‐exercise had a 15‐min washout period to ensure the cerebrovascular reactivity did not influence the next hour measures. Testing was initiated at 8:00 am and was fully completed by 7:00 pm each testing day

Seven healthy male and two female (*n* = 9) young adults (26 ± 5 years and 25 ± 4 kg/cm^2^) were recruited from the university setting. Using a randomized cross‐over cohort design, participants were tested on three separate days (control, HIIT, and MICT), with the order of all testing conditions randomized and all sessions separated by a minimum of three days. Squat–stand maneuvers were performed at baseline prior to the commencement of the test condition and again at 0, 1, 2, 4, 6, and 8 hr postexercise (Figure 1). The squats were performed for 8 hr following exercise as a conservative approach to ensure all CA measures were back to baseline. Testing began at the same time each day (8:00 am) to limit potential effects of diurnal variation (Conroy, Spielman, & Scott, 2005). Female participants were tested between days three and seven of the early follicular stage of their menstrual cycle as hormones are relatively stable during this period (Boivin & Shechter, 2010). Exclusion criteria included any history of neurological, cerebrovascular, cardiorespiratory, or chronic musculoskeletal deficits. Testing protocols were explained and demonstrated to ensure each participant was familiar and confident in performing the procedures (Claassen et al., 2015). Participants withdrew from exercise, caffeine, and alcohol for a period of at least 12 hr before each testing session (Ainslie, Barach, et al., 2007; Ainslie et al., 2008; Smirl et al., 2015, 2016). Diet across the day was controlled by having participants consume two meal replacement drinks (Vanilla Nutrition Shake, Kirkland Signature; 210 calories each) between time points two and four and two Gatorades across the day (Gatorade Perform, PepsiCo; 150 calories each). Water was provided as requested by participants.

The current data represent a subset from a larger study examining the postexercise effects of MICT and HIIT on cardiovascular (cardiac baroreceptor sensitivity and heart rate variability) and cerebrovascular metrics (neurovascular coupling, cerebrovascular reactivity, and dynamic cerebral autoregulation). Testing of each cardiovascular and cerebrovascular measure was collected in the same order at each time point, to ensure high interpretability of the data (Figure 1). The current report presents only the dynamic CA data to address the conflicting literature regarding recovery from exercise as outlined above. Written informed consent was obtained from each participant in this study, which was approved by the University of British Columbia clinical ethics review board (H16‐00507).

### 2.2 Instrumentation

Transcranial Doppler ultrasound was used to index the MCA blood velocity and the posterior cerebral artery (PCA) blood velocity by placing 2‐MHz ultrasound probes (Spencer Technologies) over the temporal acoustic windows. Once target vessels were insonated and confirmed with a visual task and a carotid artery compression (Willie et al., 2011), the probes were locked into place using a fitted head frame (Spencer Technologies). Heart rate was monitored with a three‐lead electrocardiogram (ECG) and beat‐to‐beat blood pressure was recorded using finger photoplethysmography and corrected to the height of the heart (Finometer PRO, Finapres Medical Systems, Amsterdam, Netherlands) (Omboni et al., 1993; Sammons et al., 2007). End‐tidal partial pressure of carbon dioxide (P_ET_CO_2_) was measured with an online gas analyzer (ML206, ADInstruments, Colorado Springs, CO) and was calibrated with known gas concentrations prior to data collection. Data was sampled at a frequency of 1,000 Hz (PowerLab 8/30 ML880, ADInstruments) time‐locked, and stored for offline analysis with commercially available software (LabChart version 7.1, ADInstruments).

### 2.3 Experimental protocols

**Figure 2:**
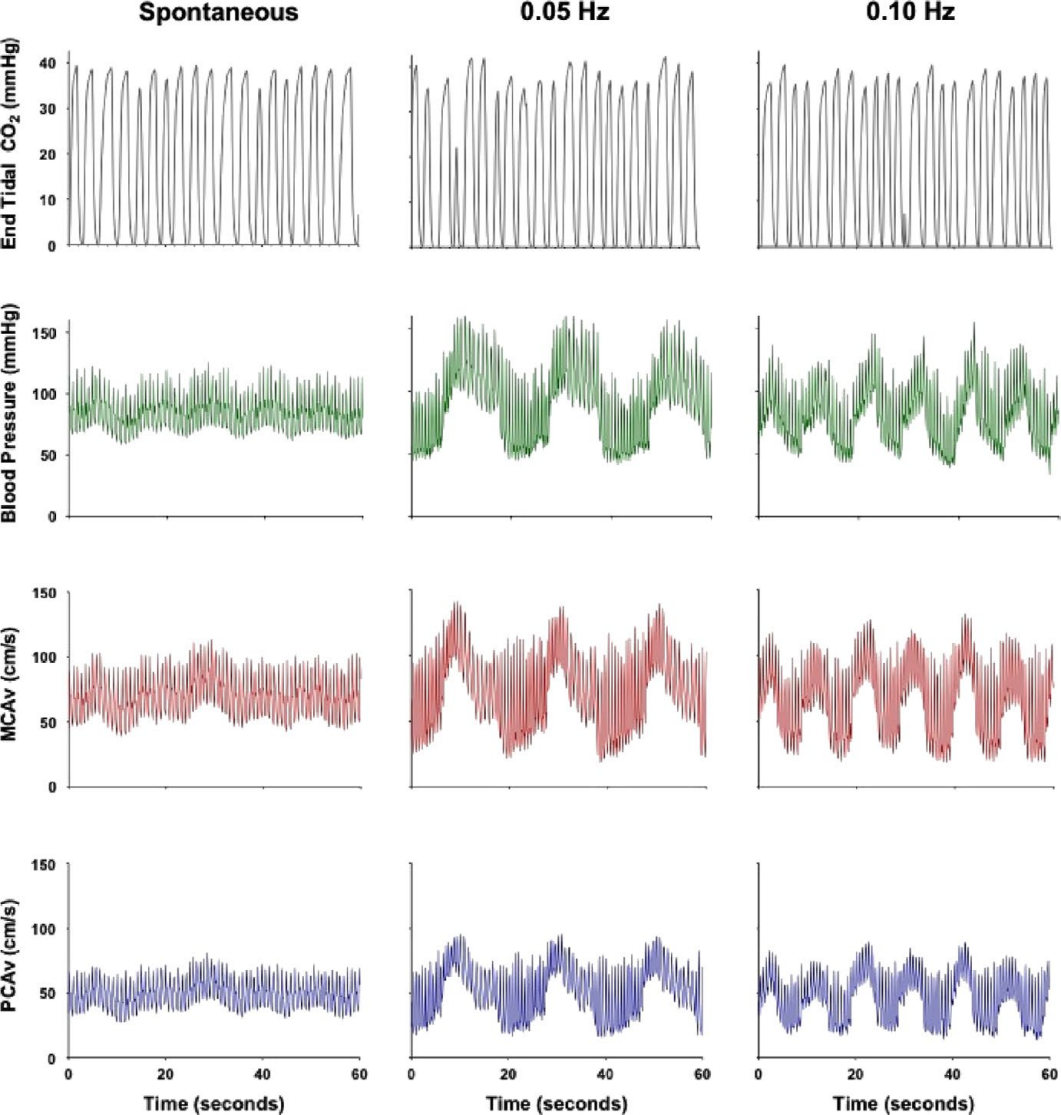
Representative trace of an individual performing the squat–stand maneuvers at frequencies of 0.05 and 0.10 Hz, with recordings of end tidal carbon dioxide values (end tidal CO_2_), blood pressure, middle cerebral artery (MCA) blood velocity, and posterior cerebral artery (PCA) blood velocity. The differential regulation between the blood pressure and MCA/PCA blood velocity traces, in particular the MCA and PCA diastolic traces show large changes analogous to the vast swings seen in the blood pressure trace, whereas the systolic aspect of the MCA and PCA traces are blunted compared to the systolic aspect of the blood pressure trace. This notion consistent with previous research (Smirl et al., 2018), which revealed systole had an augmented phase shift and a dampened normalized gain amplitude, indicative of enhanced buffering with respect to this aspect of the cardiac cycle. Additionally, this increased buffering with respect to the systolic aspect of the cardiac cycle is the underlying mechanism for the slightly reduced levels of systolic coherence values during the squat–stand maneuvers

As previously demonstrated, squad–stand maneuvers are the most reproducible method for assessing the linear aspect of the dynamic relationship between BP and CBV through transfer function analysis and thus this *gold standard* measure was used to assess dynamic CA in the current investigation (Smirl et al., 2015). To complete the squat–stand maneuvers participants began in a standing position and proceeded to squat down until the back of the knee reached ~90°. This squat position was held for a set duration before returning to the standing position. Squat–stand cycles were performed for 5 min at each of two frequencies including 0.10 Hz (5 s squatting, followed by 5 s standing) and 0.05 Hz (10 s squatting, followed by 10 s standing). Thus, the 0.05 Hz squat–stand cycle represents a frequency within the very low frequency range (0.02–0.07 Hz) of dynamic CA, whereas the 0.10 Hz squat–stand cycle produces a frequency within the low frequency range (0.07–0.20 Hz) (Claassen et al., 2015). These frequencies are within the range where dynamic CA is thought to have its greatest influence on cerebral pressure‐flow dynamics (Zhang et al., 1998). Finally, these measures were recorded in both the MCA and PCA to determine if different anatomical cerebrovascular structures are affected by exercise in a similar manner. A representative trace of the squat–stand maneuvers is presented in Figure 2.

### 2.4 Exercise protocols

As outlined above, participants were tested under three conditions—separated by at least 3 days—including HIIT, MICT, and a rest control, with both exercise protocols performed on a cycle ergometer (ergoline GmbH, Lindenstr, Germany). The HIIT protocol was broken down into a warm‐up, exercise, and cool‐down phase, lasting a combined 25 min. A 3‐min warm‐up was followed by ten 1‐min intervals of exercise to obtain a peak heart rate within 85%–90% of predicted heart rate reserve (HRR) with 1‐min active recovery periods between each minute of work at roughly 15% of the power output, consistent with previous literature (Jung, Bourne, Beauchamp, Robinson, & Little, 2015). Each individual's HRR was calculated using the Karvonen formula (Miller, Wallace, & Eggert, 1993):

$$\left[\begin{array}{c} \text{HRR}=\text{Target Intensity}\left(\text{Age Predicted Maximum Heart Rate}-\text{Resting Heart Rate}\right)\\ \;\;\;\;\;\;\;\;\;\;\;\;+\text{Resting Heart Rate} \end{array}\right]$$

Participants completed 3 min of cool down after the intervals before starting postexercise testing at hour zero. The MICT condition consisted of 45 min of exercise at a 50%–60% HRR, with a 5‐min warm‐up included at the beginning (Wewege, Berg, Ward, & Keech, 2017). Participants sat quietly for 30 min during the control condition. These exercise intensities were selected as MICT represents roughly 60% VO_2max_, which should be consistent with the peak increase in CBV (Marsden et al., 2012; Ogoh, Dalsgaard, et al., 2005), whereas the HIIT protocol produces efforts in the 80%–90% range of VO_2max_, which may lead to reductions in CBV due to hypocapnia‐induced cerebral vasoconstriction secondary to hyperventilation (Marsden et al., 2012; Ogoh & Ainslie, 2009; Ogoh, Dalsgaard, et al., 2005). Furthermore, the MICT condition reflects common athletic events such as cycling or running, in which individuals perform longer steady‐state energy outputs. Contrarily, the HIIT condition is comparable to sports such as football or hockey, in which individuals perform relatively short bursts of maximal exertion, separated by periods of rest.

### 2.5 Data processing

The R‐R intervals from the electrocardiogram were used to calculate real‐time beat‐to‐beat diastolic, mean, and systolic BP and CBV. P_ET_CO_2_ was quantified as breath‐to‐breath peak expired carbon dioxide level. All signals were visually inspected for artefacts. Data was processed and analysed using commercially available software (Ensemble‐R, Elucimed, NZ) which conforms to the standards and rigours established in the Cerebral Autoregulation Research Network (CARNet) white paper on transfer function analysis (Claassen et al., 2015). The relative power spectrum for both blood pressure and CBV was determined at the point‐estimate for the frequencies of interest (0.05 and 0.10 Hz). Transfer function analysis (TFA) coherence, phase and normalized gain values were also sampled at the point estimate within the very low frequency (0.05 Hz) and low frequency (0.10 Hz) domains. Coherence refers to the linearity of the relationship between the input (blood pressure) and output (CBV) TFA variables, which ranges from 0.0 (no relationship) to 1.0 (completely linear relationship) (Zhang et al., 1998). Phase describes the timing buffer between the input and output variables; whereas, the gain refers to the amplitude modulation within the cerebral pressure‐flow relationship (Zhang et al., 1998). Moreover, the gain metric was normalized within the current investigation to %/% to enable more appropriate comparisons presented between the various interventions in the study which resulted in alterations to either blood pressure and/or CBV. The input–output nature of the cerebral pressure–flow relationship was examined across the cardiac cycle to obtain a better understanding how each aspect is affected following a controlled bout of exercise as prior research has demonstrated there is differential CA regulation across the cardiac cycle (Ogoh, Dalsgaard, et al., 2007, 2005; Smirl et al., 2018; Wright, Smirl, Bryk, & Donkelaar, 2018). Furthermore, linear elevations to blood pressure are known to occur during increasing intensities of exercise and thus both moderate‐ and high‐intensity exercise bouts were employed to determine how these would have extended effects on CA. Finally, Phase wrap‐around was not present at any of the point estimates of the driven frequencies of interest (10 s squat–10 s stand: 0.05 Hz; and 5 s squat 5 s stand: 0.10 Hz).

### 2.6 Statistical analyses

Potential differences across conditions and time were assessed using a three (condition: HIIT, MICT, control) by seven (time: baseline, zero, one, two, four, six, eight hour follow ups) Repeated Measure ANOVA’s implemented in SPSS (Version 25.0, IBM Corp., Armonk, NY). Bonferroni‐corrected post hoc analysis was conducted to determine significant condition effects within each vessel. A priori Bonferroni‐corrected simple effects were evaluated between each postexercise time point and baseline to establish when the dynamic CA measures were no longer different from baseline. Data are presented as mean ± *SD*. Significance was set a priori at *p* < .05*.*

## 3 RESULTS

### 3.1 Cerebrovascular and cardiovascular parameters

There were no differences in CBV metrics (MCA and PCA blood velocities) at baseline between all three conditions (*p* > .129) with a good CoV reproducibility of <7%. Similarly, baseline blood pressure did not differ between conditions (*p* > .201) with CoV reproducibility of <5%. During the 30‐min control intervention P_ET_CO_2_ measures were 38 ± 1.8 mmHg, which declined to 31 ± 3.5 mmHg during HIIT, but did not change during MICT with a value of 40 ± 3.7 mmHg (Table 1). Between the control and HIIT conditions, there were no differences in MCA (63 ± 7.2 cm/s, 58 ± 11 cm/s) or PCA blood velocities (38 ± 6.3 cm/s, 36 ± 5.0 cm/s), whereas there was an increase in both during MICT (69 ± 5.6 cm s^−1^ s^−1^, 43 ± 4.1 cm/s), respectively (Table 1). Finally, heart rate measures increased from control (66 ± 7.8 bpm) to HIIT (167 ± 7.7 bpm) and MICT (136 ± 4.2 bpm); whereas, blood pressure was elevated during only during HIIT (113 ± 14 mmHg) and not MICT (109 ± 20 mmHg) compared to control (95 ± 10 mmHg).

**Table 1:** Mean Cardiorespiratory and Cerebrovascular Variables during the 30‐min control condition, 45‐min Moderate‐Intensity Continuous Training (MICT), and 25‐min High‐Intensity Interval Training (HIIT)

	Control	HIIT	MICT
P_ET_CO_2_ (mmHg)	38 ± 1.8	31 ± 3.5*	40 ± 3.7†
MCAbv (cm/s)	63 ± 7.2	58 ± 11.0	69 ± 5.6*†
PCAbv (cm/s)	38 ± 6.3	36 ± 5.0	43 ± 4.1*†
MAP (mmHg)	95 ± 10	113 ± 14*	109 ± 20
Heart Rate (bpm)	66 ± 7.8	167 ± 7.7*	136 ± 4.2*†

Note

Values are means ± standard deviation. The asterisk (*) detonates a value different from the control condition at *p* < .05. The dagger (†) detonates a value different from the HIIT condition at *p* < .05. End tidal values of carbon dioxide (P_ET_CO_2_), millimeters of mercury (mmHg), middle cerebral artery blood velocity (MCAbv), centimeters per second (cm/s), posterior cerebral artery blood velocity (PCAbv), mean arterial pressure (MAP), beats per minute (bpm).

### 3.2 Power spectrum density and coherence variation

Systolic coherence was >0.90 at all time points in the MCA and PCA at both 0.05 Hz and 0.10 Hz; whereas, all diastolic and mean coherence values were >0.96 across all time points. During the squat–stands maneuvers, the BP, MCA, PCA power spectrum densities did not differ at any time point throughout the day across all three conditions (all *p* > .196).

### 3.3 Autoregulation variations within the middle cerebral artery following exercise

**Figure 3:**
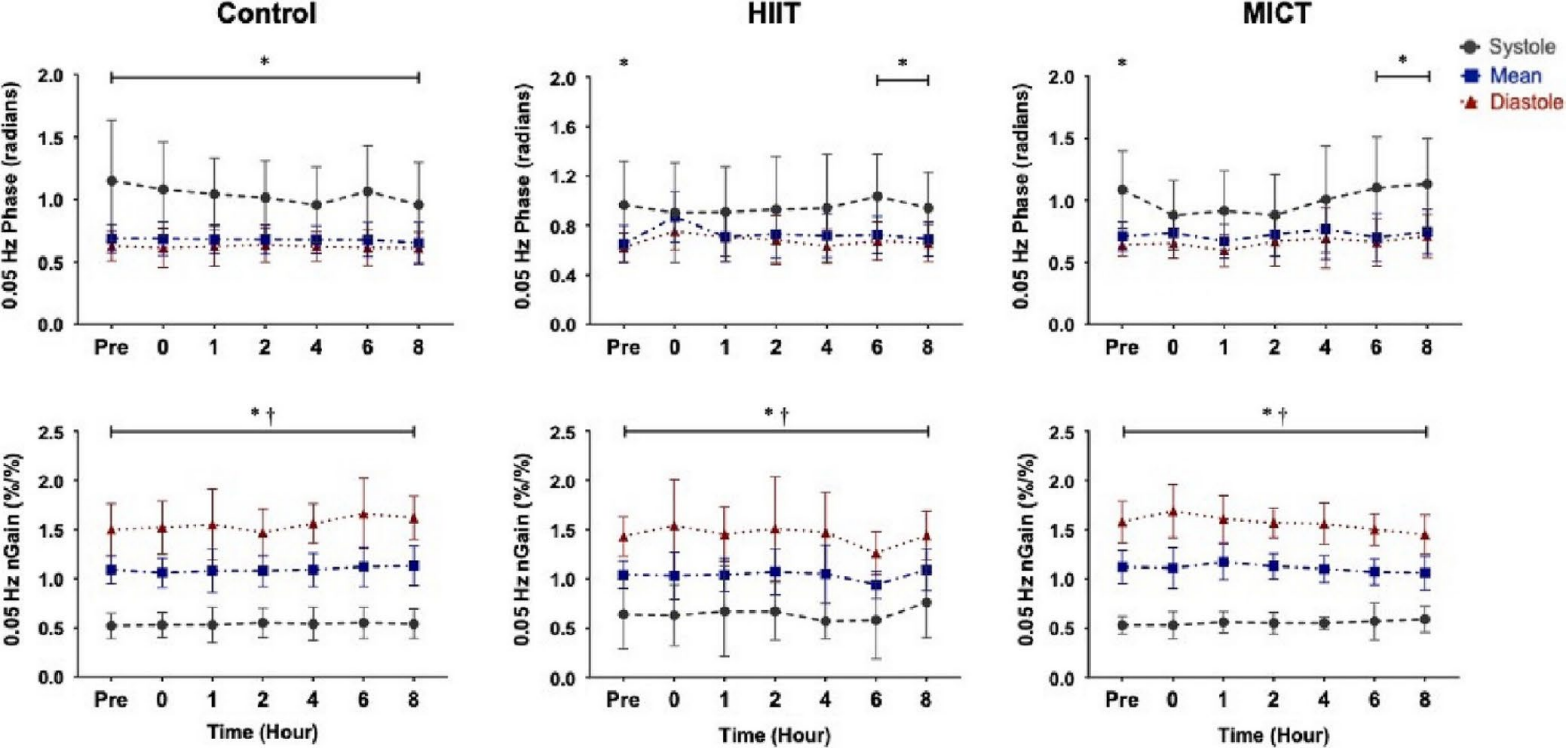
Phase and normalized gain (nGain) values within the middle cerebral artery at squat–stand maneuvers of 0.05 Hz across the cardiac cycle during control, high‐intensity interval training (HIIT), and moderate‐intensity continuous training (MICT) (*n* = 9). Data are presented as mean ± standard deviation (e.g., repeated measure analysis of variance). Asterisk (*) detonates time points where that the systolic component of the cardiac cycle is different than mean and diastole (*p* < .05) and dagger (†) detonates time points where mean is different than diastole (*p* < .05). The grey (dashed), blue (dashed‐dotted), and red (dotted) data points represent the systolic, mean, and diastolic components of the cardiac cycle, respectively

**Figure 4:**
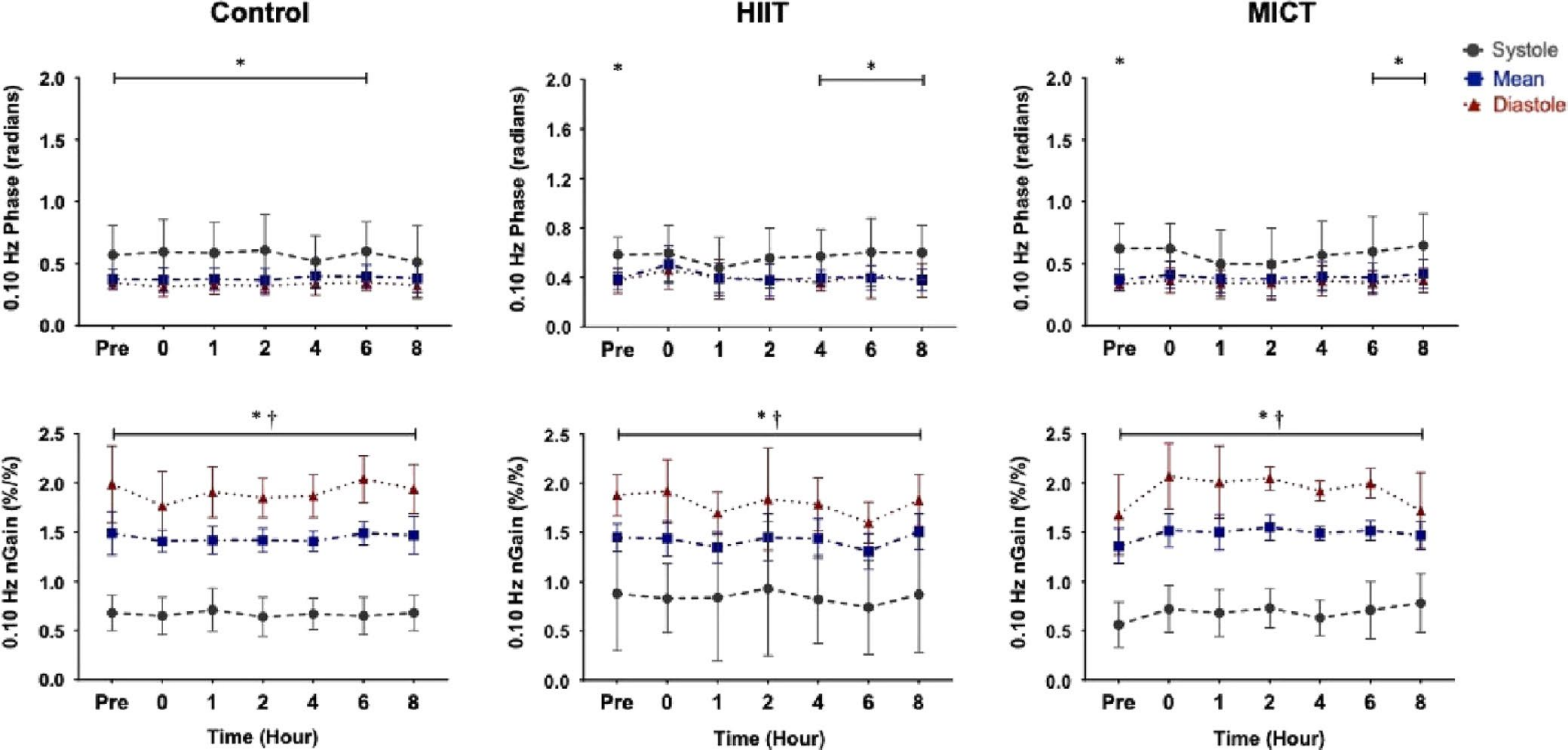
Phase and normalized gain (nGain) values within the middle cerebral artery at squat–stand maneuvers of 0.10 Hz across the cardiac cycle during the control, high‐intensity interval condition (HIIT), and moderate‐intensity continuous training (MICT) conditions (*n* = 9). Data are presented as mean with stand deviation (e.g., repeated measure analysis of variance). Asterisk (*) detonates a value where systole was different than the mean and diastolic values (*p* < .05) and dagger (†) detonates where mean is different than diastole (*p* < .05). Systole, mean, and diastole are represented by the grey (dashed), blue (dash‐dotted), and red (dotted) data points, respectively

At baseline for all three conditions, there was differential regulation for systolic compared to the mean and diastolic components (all *p* < .012*;* Figures 3 and 4). During the control condition, systolic MCA phase at both frequencies was different from mean and diastolic values across each time point (all *p* < .050), aside from an anomaly recorded at hour eight, where the systolic MCA 0.05 Hz phase was similar to the other components (*p* = .148; Figure 4). Following HIIT, the difference in phase regulation between systole and diastole/mean, was absent from hour zero until hour four at 0.05 Hz (all *p* > .102; Figure 3) and from hour zero until hour two at 0.10 Hz (all *p* > .110; Figure 4). This known differential regulatory aspect of CA across the cardiac cycle returned back to significance by hours six and eight at 0.05 Hz (all *p* < .022; Figure 3) and four to eight at 0.10 Hz (all *p* < .015; Figure 4). Similar to the HIIT condition, the follow‐up measures after the MICT protocol demonstrated the phase metric across the cardiac cycle was comparable between systole, mean and diastole within the MCA from hours zero to four within both frequencies (all *p* > .079; Figure 3), returning to the expected separation by hours six and eight (all *p* < .030; Figure 4).

In contrast to the findings in the phase metric, the systolic aspect of normalized gain within the MCA, was different from mean and diastolic values at every time point across all three conditions at both 0.05 and 0.10 Hz (all *p* < .005; Figures 3 and 4). Concurringly, all mean and diastolic normalized gain metrics were different across all conditions at both squat–stand frequencies (all *p* < .001; Figures 3 and 4).

### 3.4 Autoregulation variations within the posterior cerebral artery following exercise

**Figure 5:**
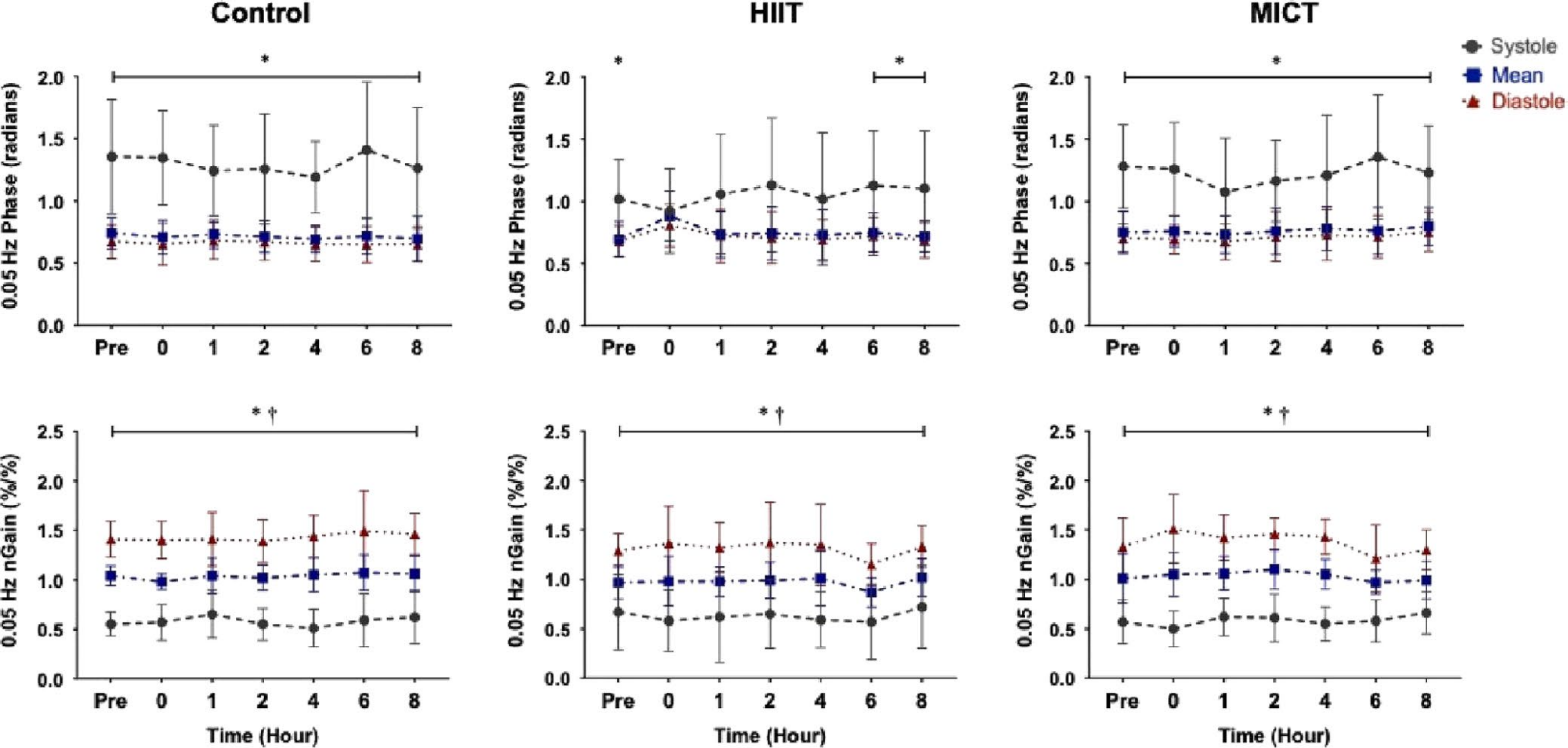
Phase and normalized gain (nGain) values within the posterior cerebral artery across the cardiac cycle during control, high‐intensity interval training (HIIT), and moderate‐intensity continuous training (MICT) at squat–stand maneuvers of 0.05 Hz (*n* = 9). Data are presented as mean ± standard deviation (e.g., repeated measure analysis of variance). Asterisk (*) detonates a time point where diastole and mean were different than the systolic component (*p* < .05) and dagger (†) detonates time points where mean is different than diastole (*p* < .05). Systolic data is represented in grey (dashed), mean in blue (dash‐dotted), and diastole in red (dotted)

**Figure 6:**
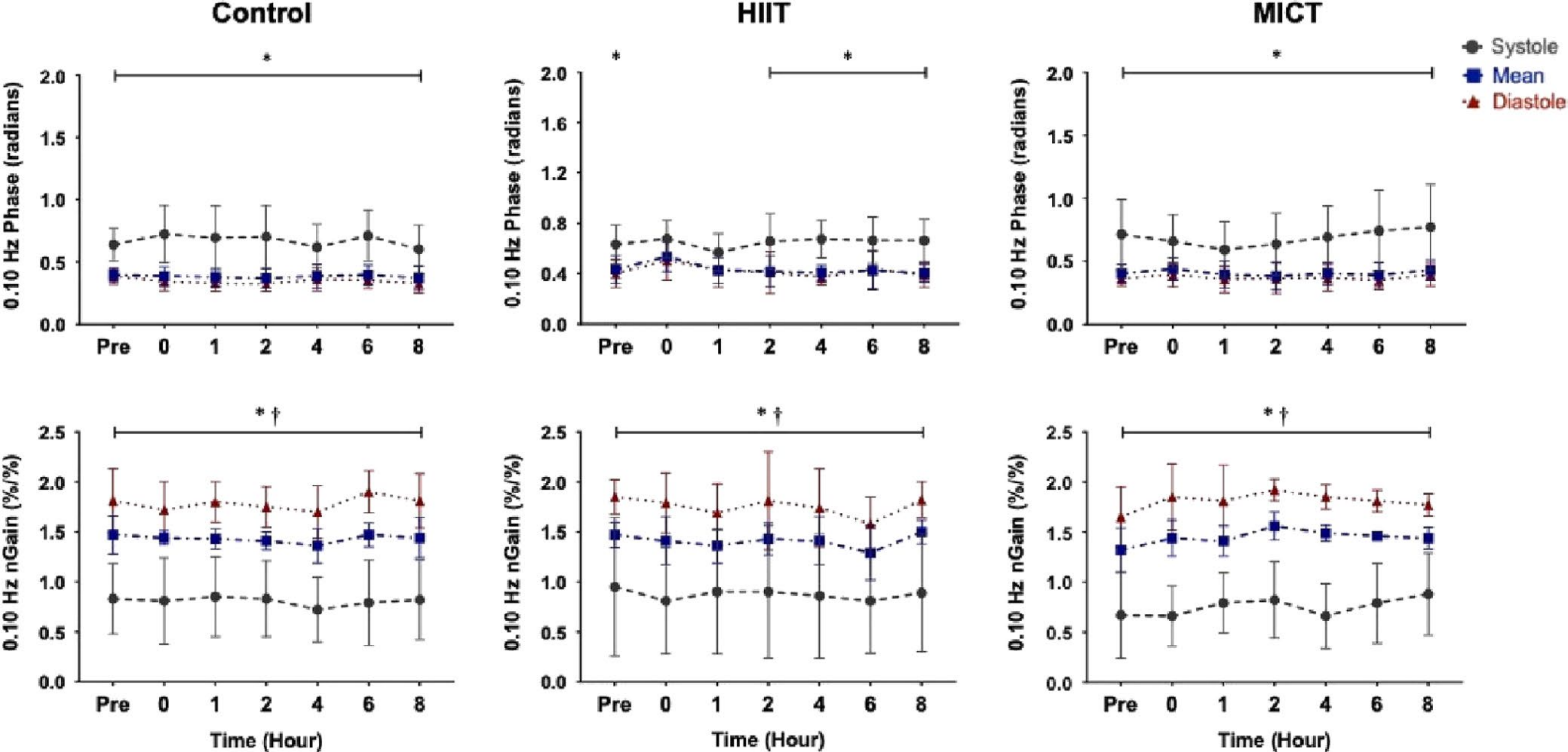
Mean and standard deviation phase and normalized gain (nGain) values at squat–stand maneuvers of 0.10 Hz within the posterior cerebral artery across the cardiac cycle in control, high‐intensity interval training (HIIT), and moderate‐intensity continuous training (MICT) (*n* = 9). Data are represented as mean ± standard deviation (e.g., repeated measure analysis of variance). Asterisk (*) detonates a time point where the systolic value is different than the diastolic and mean components (*p* < .05) and dagger (†) detonates a tome point where the mean values is different than diastole (*p* < .05). Systole is shown in grey (dashed), mean in blue (dashed‐dotted), and diastole in red (dotted)

Within the PCA, systolic phase was different than mean and diastolic components across the control day (all *p* < .001) and between the three baseline conditions (Figures 5 and 6). However, following HIIT the PCA diastolic and mean were not different from systole from hours zero to four at 0.05 Hz (*p* > .050; Figure 5) and at hours zero and one for 0.10 Hz (all *p* > .055; Figure 6). The differential in regulation returned at hour two (*p* = .015) and hour six (*p* = .014) for both 0.05 Hz and 0.10 Hz, respectively, and stayed significant until hour eight for both frequencies (all *p* < .015; Figures 5 and 6). Similar to control, the systolic PCA phase varied compared to mean and diastole at each time point in both frequencies across the day for the MICT condition (all *p* < .017; Figures 5 and 6).

Further, the PCA systolic normalized gain values were divergent compared to the mean and diastolic values at 0.05 and 0.10 Hz across the control, MICT, and HIIT conditions (all *p* < .050; Figures 5 and 6). Lastly, the normalized gain values were different between mean and diastole at 0.05 and 0.10 Hz across all conditions (all *p* < .001*;* Figures 5 and 6).

## 4 DISCUSSION

The key findings from this investigation were as follows: (a) the differential in autoregulation across the cardiac cycle was diminished immediately following both MICT and HIIT exercise protocols; (b) at baseline, the systolic component of the cardiac cycle regulated differently than the mean and diastolic components, but this disappeared for up to 4 hr following exercise; (c) all CA metrics had returned to the expected divergent systolic regulation by 6 hr following exercise, and was comparable to pre‐exercise baseline levels; (d) dynamic CA assessed using TFA and driven BP oscillations yields high within‐individual reproducibility across the day. Together, these findings support our hypotheses that CA is impaired following exercise, however the duration CA was impaired was longer than hypothesized. Moreover, it is pertinent to assess each component of the cardiac cycle independently to obtain a comprehensive understanding of the cerebral–pressure flow relationship, as exercise was shown to differential affect each component. Furthermore, the speculative 12–24 hr time constraint typically used in the literature is overly conservative and this restrictive testing window can be reduced to 6 hr, which may have significant implications for future study designs evaluating cerebrovascular function in regularly active individuals (e.g., competitive athletes) using driven methodology.

### 4.1 Comparison with previous studies

Previous studies examining CA during recovery from exercise have produced equivocal findings with several studies finding dynamic CA remains intact (Murrell et al., 2009, 2007; Tsukamoto et al., 2019; Willie et al., 2013); in contrast, Ogoh and coworkers (Ogoh, Fisher, et al., 2007) reported impairments in TFA‐derived metrics of CA. However, the previous studies listed were based upon cerebrovascular resistance and cerebrovascular conductance calculations, bolus injections, or spontaneous TFA methodology to quantify CA, as opposed to using driven BP oscillations to enhance reproducibility as was done in the current investigation. Ogoh and colleagues (2007) noted an increase in low frequency diastolic gain immediately postexercise at 1–3 and 6–8 min into recovery. This finding extends previous work demonstrating increases in diastolic gain *during* exhaustive exercise, while the mean and systolic components were not affected, indicating each phase of the cardiac cycle is differentially regulated following exercise (Ogoh, Dalsgaard, et al., 2005). Furthermore, previous research conducted by Smirl et al. (2018) has established that the mean and diastolic phase components of CA operate in a similar manner, whereas the systolic component exhibits greater pressure buffering. However, as the current results demonstrate, both MICT and HIIT resulted in a reduced systolic phase within the MCA up to 4 hr postexercise, suggesting relatively less effective pressure buffering. Contrastingly, phase was impacted in the PCA up to 4 hr only following HIIT, whereas MICT induced limited effect on CA in the PCA. Moreover, in contrast to Ogoh and colleagues (Ogoh, Dalsgaard, et al., 2005; Ogoh, Fisher, et al., 2007), no alterations were observed in normalized gain following exercise at either frequency. One possible explanation for the difference in pressure regulation across the cardiac cycle involves the gradual withdrawal of the exercise‐induced sympathoexcitation, which has been demonstrated to be related to CA impairments (Ainslie, Barach, et al., 2007; Ogoh, Dalsgaard, et al., 2005). Additionally, an increase in cerebral metabolites (i.e., epinephrine, norepinephrine, prostaglandins, brain‐derived neurotropic factor, etc.) may build up throughout the exercise bout which, in turn, could have altered the cerebrovasculature immediately during the postexercise period (Walsh & Tschakovsky, 2018; Zouhal, Jacob, Delamarche, & Gratas‐Delamarche, 2008). It is possible these responses could (at least in part) explain the altered 0.10 Hz phase metrics in both vessels, which persisted to the 4th hr of recovery (Figures 3, 4, 5, 6). However, the mechanistic underpinnings of the observed CA responses were not directly examined in the current study, though further research is warranted toward this end.

Investigations have also shown CA remains stable following 40 min of cycling exercise at 60% VO_2max_ (Willie et al., 2013) and after running a marathon (Murrell et al., 2009, 2007). One important consideration when comparing previous moderate exercise studies to the current investigation is they were performed with spontaneous (instead of driven) oscillations in BP; as such their phase and gain results are based on lower coherence values, which have been shown to limit reproducibility, thereby impacting the interpretation and reliability of the reported findings. Conversely, this investigation employed the *gold standard* squat–stand maneuvers for assessing TFA linearity and observed uniform values in phase across the cardiac cycle at the 0.05 Hz and 0.10 Hz frequency for a maximum of 4 hr postexercise (Smirl et al., 2015). Koch et al. (2005) also observed a decrease in phase following dynamic resistance exercise during the early recovery period (80 s). The change in phase angle across the cardiac cycle in the current study following MICT exercise may be attributed to more compliancy within the dilated vasculature, known to occur during moderate exercise (Marsden et al., 2012; Ogoh & Ainslie, 2009, 2009; Smith & Ainslie, 2017). Such dilation likely occurred throughout the entirety of the MICT protocol, due to hyperpnea‐induced vasodilation, which potential led to more compliant vasculature. This is demonstrated in Table 1 where MCA and PCA blood velocity were higher during MICT exercise compared to the control condition. This would result in a reduced buffering capacity of the vessels, due to the dilated vasculature being less compliant to surges in blood flow. As the diastolic component of the cardiac cycle has a more pressure‐passive nature, this stiffening would have a greater impact on the systolic component of the cardiac cycle (Smirl et al., 2018). This is shown in Figures 3 and 4 where the differential in systolic phase regulation in general did not return back to baseline values up to hour six, whereas diastolic and mean values increase slightly at hour zero following exercise, but generally return to their respective values at roughly hour one.

Finally, there were no differences in dynamic CA metrics across the control day, demonstrating driven oscillations yield high within‐day reproducibility estimates. This is in contrast to a previous study, which found CA was impaired in the morning (6:00–8:00 am) compared to the evening (6:00–8:00 pm) (Cummings, Swart, & Ainslie, 2007). However, this may be due to two methodological differences. First, Ainslie and colleagues (Cummings et al., 2007) examined CA within the first 30 min upon waking, where reductions in endothelial function may have been present, whereas our first assessment was performed at least 1 hr after the participant awoke. Second, our study used squat–stand maneuvers, as opposed to the thigh cuff inflation–deflation method used by Cummings and colleagues (Cummings et al., 2007). Although thigh cuff inflation–deflation methods increase coherence values relative to spontaneous measures, squat–stand maneuvers augments coherence to near linear levels, which reduces the amount of variation within TFA measures. Although further exploration is warranted, our results demonstrate highly reproducible CA estimates when using squat–stand maneuvers and TFA‐based analytic techniques across the typical workday. It seems likely that squat–stand maneuvers elicited stronger hemodynamic changes (~30–40 mmHg), which in turn resulted in a parallel increase in CBV (Smirl et al., 2015). These large swings in hemodynamic and cerebrovascular variables is the underlying mechanism for the increase in linearity within the cerebral‐pressure flow relationship and the substantially high coherence values (>90%) across the cardiac cycle. Moreover, despite known diurnal changes to CBV (Conroy et al., 2005) and blood pressure (Degaute, Van De Borne, Linkowski, & Van Cauter, 1991; Kawano, 2011), dynamic CA remained stable across the day. This may be attributable to the fact autoregulation plays a considerable role in safeguarding against strokes and cerebrovascular accidents, and therefore despite known changes to CBV and blood pressure across the day, CA appears to remain intact (Figures 3, 4, 5, 6) (Jordan & Powers, 2012). However, further research is needed to understand the exact mechanistic reasons as to why this is driven CA metrics are consistent across the day while other cardiovascular parameters have shown diurnal effects.

### 4.2 Potential mechanisms underlying the alterations between exercise conditions

During the MICT condition, both MCA and PCA blood velocities were elevated compared to control, however P_ET_CO_2_, remained similar (Table 1). Contrarily, CBV in the MCA and PCA did not change during exercise relative to the control, but P_ET_CO_2_, was attenuated (Table 1). Heart rate was elevated during both exercise interventions; whereas, blood pressure was only raised during HIIT (Table 1). The latter finding could be derived to the fact that the MICT condition was calculated to occur at roughly 50%–60% VO2max, which was insufficient to reach significance from baseline values, but was sufficient to elicit the expected elevations to CBV (Table 1). Collectively, the physiological changes seen during the three conditions employed in this investigation are in agreement with previous work that have examined physiological measurements during exercise (Ogoh & Ainslie, 2009; Smith & Ainslie, 2017). Furthermore, individuals are able to maintain exercise up to an intensity of roughly 60%–70% of their VO_2max_ for prolonged durations as this intensity is typically below the anaerobic threshold (Mateika & Duffin, 1995). Therefore, there is an equivalent rise in both oxygen consumption and carbon dioxide production, where the surge in carbon dioxide in the bloodstream (indexed with P_ET_CO_2_) increases CBV, causing cerebrovasculature dilation (Marsden et al., 2012; Mateika & Duffin, 1995; Ogoh & Ainslie, 2009). Once the anaerobic threshold is surpassed, an individual will shift from aerobic metabolism to anaerobic metabolism, causing acidity to increase (Beaver, Wasserman, & Whipp, 1986). The body counteracts this acidotic stimulus by increasing ventilation to buffer against the drop in pH (Beaver et al., 1986; Ogoh, Dalsgaard, et al., 2005). An increased respiration rate at higher exercise intensities (RER > 1.0) leads to a reduction in P_ET_CO_2_, which is the primary driver of hyperventilation‐induced cerebral vasoconstriction (Marsden et al., 2012). The data collected during each condition shown in Table 1 demonstrates, these effects were achieved with both moderate and high intensity exercise bouts. Although, neither end‐tidal values during exercise were different from control, CBV increased during MICT demonstrating cerebral vasodilation likely occurred due to the aforementioned reasons. Further, CBV was similar during HIIT compared to control, demonstrating the lower end‐tidal values led to slight cerebral vasoconstriction.

Moreover, at 60% of VO_2max_ sympathetic activity has been shown to increase by 24%, whereas at 75% of VO_2max_ it is elevated 79% (Saito & Nakamura, 1995). Therefore, the higher intensity of exercise employed in this study (~85%–90% VO_2max_) would have increased the total amount of sympathetic nervous activity, resulting in further increases in cerebral vasoconstriction (Karemaker, 2017). Coinciding with the augmented sympathetic activity there is a corresponding decrease in parasympathetic activity, which enables even more constriction to occur within the cerebrovasculature (Karemaker, 2017). Combined, these mechanisms have been theorized to be an important factor regulating the permeability of the blood‐brain barrier, especially when CA is challenged (Ogoh, Fadel, et al., 2005). However, sympathetically mediated cerebral vasoconstriction is expected to decline immediately following exercise, potentially explaining the alterations in phase immediately following the HIIT condition. Furthermore, exhaustive exercise increases the presence of ammonia circulating within the vasculature, which has been demonstrated to impair dynamic CA and may thus provide an additional mechanism responsible for the more extensive alterations observed to the high‐intensity form of exercise in the current investigation (Bachmann, 2002; Ogoh, Dalsgaard, et al., 2005).

To prevent BP surges from causing irreversible damage in the cerebrovasculature, CA is thought to act as a buffer to protect the brain from cerebral hyperperfusion (Ogoh, Fisher, et al., 2007; Smirl et al., 2018; Wright, Smirl, Bryk, & Donkelaar, 2018). Ogoh and coworkers (Ogoh, Dalsgaard, et al., 2005; Ogoh, Fisher, et al., 2007) found diastolic dynamic CA was impaired during exercise and acute recovery (10 min) within the LF band, despite the 5‐min averaged diastolic MCA blood velocity appearing to be well maintained. These results are similar to those previously reported in athletes who have sustained an acute sports‐related concussion (Wright, Smirl, Bryk, & Donkelaar, 2018). Both MCA and PCA blood velocities have previously been shown to increase following moderate exercise, likely influenced by the elevated P_ET_CO2 associated with subanaerobic threshold exercise. This stimulus would lead to a smaller dilatory reserve within the vessels, as the vasculature would be closer to maximal dilation, thereby reducing the capacity for further dilation with additional stimuli to increase blood flow. In turn, this would be reflected as a reduction in buffering capacity as reduced phase, which was more prevalent within the systolic component. Contrarily, the high‐intensity intervals likely encouraged cerebral vasoconstriction via the effects of compensatory hyperventilation, which would have also led to a stiffer vasculature through increased vessel tone. In turn, dilatory capacity may be diminished with stimulus to increase blood flow. Ultimately, both exercise protocols impaired the pressure buffering capacity of the cerebrovasculature in a divergent manner. Willie and colleagues (2012) previously demonstrated regional differences in cerebrovascular reactivity to carbon dioxide between anterior and posterior circulatory beds, suggesting this may have been due to the relative proximity of the posterior circulation to the medulla oblongata, which contains the respiratory and cardiovascular control centers. Within the current investigation, CA in the MCA was impacted to a greater extent than in the PCA, perhaps for similar reasons. However, this was not directly examined within this investigation and therefore, future studies should attempt to discern the reasons for differences in CA between the posterior and anterior circulations.

### 4.3 Between day reproducibility of dynamic cerebral autoregulation metrics

There are several ways to assess CA, however induced BP oscillations are preferable to those that occur spontaneously, as the former increases the input–output relationship, ultimately leading to better interpretability of TFA metrics (Claassen et al., 2009, 2015; Simpson & Claassen, 2018; Smirl et al., 2015). Driven oscillations in BP during exercise have been successfully performed through the use of oscillatory lower body negative pressure (OLNBP) which elicits similar surges in BP as traditionally observed with squat–stand maneuvers (Smirl et al., 2015). However, without the elevated cardiac output induced by exercise, OLBNP under resting conditions was unable to replicate the reproducibility achieved with squat–stand maneuvers at various frequencies within the CA range (Smirl et al., 2015). The marked elevation in TFA metric linearity is responsible for the low within‐subject between‐day CoV (<13%) in all dynamic CA measures.

### 4.4 Implications for future dynamic cerebral autoregulation assessments

The findings of this study will help guide future CA research, as it provides researchers with objective empirical evidence regarding the timeframe for which subjects are required to refrain from exercise (6 hr) prior to data collection when employing driven methodology. Furthermore, the modality of exercise performed by individuals has appreciable effects on the cerebral pressure‐flow response. As such, future investigations will need to also consider the modality of exercise performed and potentially the sporting background (Perry et al., 2019) of subjects as the potential long‐term exposure to MICT and/or HIIT patterns may differentially alter cerebral autoregulatory capacity (Drapeau et al., 2019; Labrecque et al., 2019; Lind‐Holst et al., 2011). Taken together, the results from this investigation demonstrate that refraining from exercise for 12–24 hr prior to dynamic CA data collection is excessively conservative. Rather, the minimum time of 6 hr suggested by our results would allow for more opportunities to engage in research opportunities with athletes and other regularly active individuals who participate in practices, games, or training sessions daily. Finally, the results show when dynamic CA is quantified using squat–stand maneuvers, there is a high‐degree of within‐day reproducibility across the typical workday.

### 4.5 Limitations

A drawback of using transcranial Doppler ultrasound to measure CA it is only capable of quantifying velocity and is unable to measure flow. However, assuming velocity is equivalent to flow with the presumption the diameter of the vessel being insonated does not change, one is theoretically able to index CBV. It has been demonstrated when P_ET_CO_2_ is within eight mmHg of eucapnia, the diameter of cerebral arteries remains relatively constant which was examined through the use of high‐resolution magnetic resonance imaging (Coverdale, Gati, Opalevych, Perrotta, & Shoemaker, 2014; Verbree et al., 2014). In the present investigation, all squat–stand protocols were performed at eucapnia, supporting the data collected is an accurate representative index of CBV and was not influenced by changes in carbon dioxide. Additionally, a draw back to the methods employed in this study was HRR was derived based upon the common formula for maximal HR (220‐age) and was not precisely quantifying each person's VO_2max_, to ensure the exercise bouts were performed below the anaerobic threshold. Furthermore, this was only examined following aerobic/endurance exercise and did not include resistance exercise. Therefore, future studies should examine the recovery‐time course following resistance activities as they may cause different acute hemodynamic/physiological responses. Additionally, a drawback of this investigation is the small sample size (*n* = 9), partially attributed to the time burden required for each participant to complete the study. However, a randomized cross‐over design was utilized in this investigation to allow each individual to be their own control across each condition. This is a strength of the methodology as this substantially reduces the likelihood any confounding effects may influence the results due to a small number of subjects, such as history of concussion/subconcussive impacts (Wright et al., 2018), cardiorespiratory fitness levels (Labrecque et al., 2017), history of migraines (Knol et al., 2020), and mood disorders (Toma, MacIntosh, Swardfager, & Goldstein, 2018), etc. Therefore, despite the smaller sample size, the results are highly valid, as there is a reduced likelihood that any extraneous variables may influence the results given the methodology employed. Moreover, the sample size of this investigation is directly proportionate to several other published studies examining CA (Aaslid et al., 1989; Ainslie et al., 2008; Bailey et al., 2013; Claassen et al., 2009; Ogoh, Dalsgaard, et al., 2005; Ogoh, Nakahara, Ainslie, & Miyamoto, 2010; Smirl, Haykowsky, et al., 2014; Smirl et al., 2015, 2016; Tsukamoto et al., 2019). Finally, as the population selected to participate in this study were young, physically active university students, the current results may not be directly generalizable to clinical and elderly populations.

## 5 CONCLUSIONS

In summary, exercise was shown to affect dynamic CA into recovery up to 4 hr following exercise, with HIIT demonstrating greater affects than MICT. Furthermore, each phase of the cardiac cycle responded differently to exercise, with the systolic component showing impairments for up to 4 hr, compared to diastolic and mean which recovered after 2 hr in all measures. This is indicative of decreased ability of the vasculature to buffer due to more compliancy from hyperpnea following moderate exercise, which was evident in the MICT condition as the typical differential phase regulation seen between systole and mean/diastole was removed immediately following MICT and returned at hour six. Similarly, this occurred succeeding the HIIT protocol as the phase divergent regulation disappeared following HIIT, and reappeared at hour six; however, this was the resultant of stiffer vasculature due to increased vessel tone following high intensity exercise. Therefore, it is imperative for future studies examining driven dynamic CA measures to test participants across the cardiac cycle a minimum of 6 hr following exercise, irrespective of the intensity. These findings substantially reduce the common practice within literature of mandating participants to abstain from exercise for 12–24 hr. Finally, dynamic CA metrics did not vary at any time point across the control day, allowing future studies to reliably assess these measures using the squat‐stand protocols across the typical workday (8:00 am to 7:00 pm).

## CONFLICT OF INTEREST

None declared.
